# Exploring Plant Agro‐Industrial By‐Products as a Source of Fibrous Food Ingredients: A Review of Extraction Methods and Technological Properties

**DOI:** 10.1111/1750-3841.70408

**Published:** 2025-07-12

**Authors:** Raquel Martins da Silva Fernandes de Oliveira, Herald Martinho Lino dos Santos, Sibele Santos Fernandes, Mariana Buranelo Egea

**Affiliations:** ^1^ Agronomy Department, Agronomy School Federal University of Goiás (UFG) Goiânia Brazil; ^2^ Goiano Federal Institute Campus Rio Verde Rio Verde Goiás Brazil; ^3^ School of Chemistry and Food Federal University of Rio Grande Rio Grande Brazil

**Keywords:** agro‐industrial by‐products, fibrous extraction, fibrous ingredients, plant‐based ingredients

## Abstract

The agro‐industrial sector generates a large amount of by‐products, and researchers have been seeking new ways to use them, especially those of plant origin. The concept of biorefinery and circular bioeconomy aims to enhance and maximize the use of organic ingredients, emphasizing the use of renewable and sustainable materials. Many by‐products are rich in both macro and micronutrients, making them attractive for reuse and the development of new products. Plant fibers, composed of sclerenchyma cells, are an important example and are classified according to their origin. The use of food processing by‐products as a source of fiber has great potential to reduce waste and improve nutritional value. Industrial interest in plant fibers is growing, making them a viable alternative to traditional ingredients. These fibers, originating from agro‐industrial by‐products, have proven profitable for food enrichment. This review examines the current state of the art in recovering and applying fibrous by‐products, highlighting methods for obtaining them, their technological properties, and potential future applications. Furthermore, the conditions for modifying agro‐industrial waste are analyzed, and the importance of understanding the original fibrous matrix is emphasized to select the most suitable transformation process, thereby ensuring a high‐quality final product.

## 1 Introduction

The search for natural and renewable resources is growing, driving research to reuse by‐products in various technological areas and promote the circular economy. Global primary crop production has grown by 3% since 2022 and by 27% compared to 2010 (Fuglie et al. 2024). Globally, more than 1 billion tonnes of agro‐industrial by‐products are generated annually. Still, less than 85% are used (Souza et al. 2022), and predominantly rich‐fiber by‐products, such as peels and seeds, among others (A. C. Lemes et al. 2021).

Plant fibers are established as sclerenchymatic cells that are elongated and, generally, with thick walls. They can also be defined as an agglomeration of cells considered structural components of plants, which can be divided into smaller groups based on their origin (Feng et al. 2024). The composition of dietary fiber varies depending on its origin, being defined as remains of edible parts of a plant or analogous carbohydrates, consisting of carbohydrate polymers, non‐starch polysaccharides, and lignin, which are resistant to digestion, complete and/or partial fermentation by human digestive enzymes, and absorption in the intestine human (Sztupecki et al. 2023).

**FIGURE 1:**
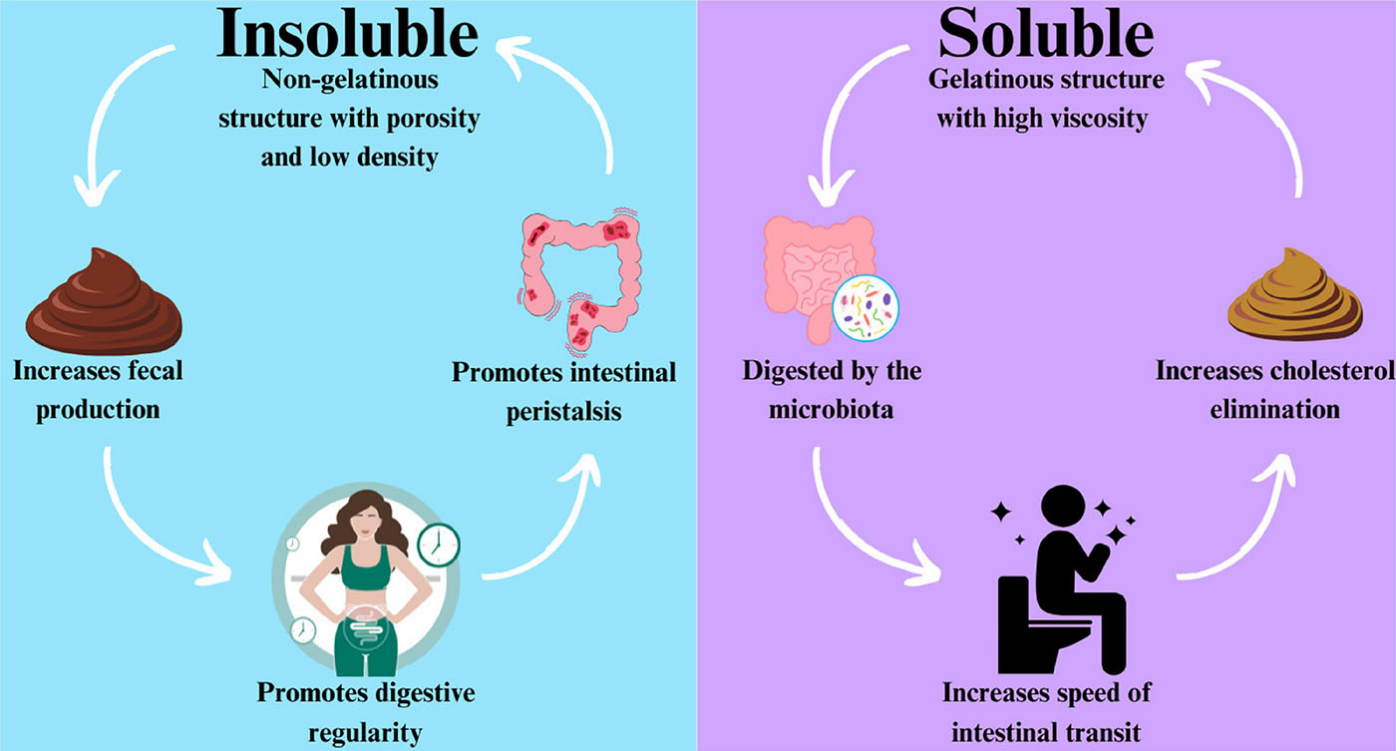
A diagram showing the characteristics and actions of insoluble and soluble fibers.

Fibers are classified as soluble or insoluble according to their solubility. Insoluble fibers (Figure 1) do not dissolve in water and remain almost unchanged after digestion (they do not contribute to the mixture's viscosity). Furthermore, they have a non‐gelatinous structure and are derived from the structural parts of plants (such as shells, seeds, and stalks) or whole cereals or grains. Insoluble fibers present porosity, low density, the ability to increase fecal production, promote intestinal peristalsis, have limited fermentation, and promote digestive regularity (Tang et al. 2022).

Soluble fibers increase the fecal volume, impacting the speed of intestinal transit and, due to their ability to absorb water, forming viscous gels that can capture cholesterol, preventing its reabsorption into the bloodstream (Figure 1). Once the soluble fiber gel is formed, there is a delay in the absorption of nutrients and gastric emptying, contributing to increased satiety and therefore weight loss (Gill et al. 2021). Furthermore, soluble fiber plays a crucial role in the intestinal microbiota, as it is readily fermented there, producing metabolites considered essential for human health (Baky et al. 2024).

Utilizing by‐products to produce ingredients with varying proportions of soluble and insoluble fibers present a sustainable and innovative alternative in the food industry (Marczak and Mendes 2024). Raw materials such as peel, seed, and pulp can be concentrated or modified to alter the fibers present, creating ingredients with distinct nutritional profiles. Produce different ingredients from waste, allowing the creation of food products that meet different dietary needs, such as foods rich in soluble fiber, which helps regulate cholesterol and glycemic control, or products with a higher concentration of insoluble fiber, which favors intestinal function (Iqbal et al. 2022). Furthermore, when an ingredient with different proportions of soluble and insoluble fibers are produced and introduced into processed foods, it is expected that its sensory contribution, especially texture, will depend on the characteristics of these ingredients (Marczak and Mendes 2024). In addition to adding value to by‐products, this approach reduces waste, promoting more efficient and eco‐friendly practices in the food production chain (Lemes et al. 2022). Therefore, this review presents the state‐of‐the‐art regarding the recovery and utilization of fibrous food by‐products from agro‐industrial waste, focusing on methods of obtaining these products, their technological properties, and potential future food applications.

The research was conducted in the SCIELO, ScienceDirect, SCOPUS, and Springer databases, focusing on studies published between 2019 and 2025. The descriptors used were: “plant fiber” OR “plant fibre” AND “extraction” OR “modification” AND “ functional ” OR “ technologies ” OR “techno‐functional” AND “by‐product”. The inclusion criteria were manuscripts that, based on the title and abstract, made it possible to identify that the study had applied some method of modification to plant material and were published from 2019 onwards. At least two authors reviewed all studies.

## 2 Generation and Use of Agro‐Industrial By‐Products

Due to increased demands, the agricultural movement is under pressure to become more productive, often overlooking or ignoring the negative aspects and environmental impacts caused by the relentless pursuit of high yields. The by‐products generated are often dumped in landfills and/or burned to avoid microbial agglomerations. Still, they cause environmental changes (Ben‐Othman et al. 2020) and can be underused in agriculture or animal feed (A. C. Lemes et al. 2021).

The use of by‐products as raw materials for other purposes, as envisaged by the circular economy, is considered a strategy to reduce negative environmental impacts related to the entire production chain, in addition to combating hunger, a critical point highlighted in the Sustainable Development Goals (SDGs) (Barros et al. 2020). The proposal to use agro‐industrial plant by‐products is plausible, given that the annual generation of global agro‐industrial inputs approaches 140 billion tons, with 2.96 billion tons relevant to the food industry (Tripathi et al. 2019).

The circular bioeconomy concept aims to value and maximize the use of by‐products as a whole (Raj et al. 2022), harnessing all available energy (chemical, electrical, and thermal) in certain materials to reduce the dependence on non‐renewable resources. Although the valorization of agro‐industrial by‐products as an ingredient is potentially viable as a simple alternative, this strategy requires careful application, especially regarding the microbiological issues involved (Kumar et al. 2023; A. C. Lemes et al. 2021).

The most used approaches in attempts to manage inputs are based on the use of plant by‐products to supplement animal feed (in particular, by‐products rich in fiber) or the use of bioprocesses to obtain biofuels (Sun et al. 2024). The market still demands studies in new areas, particularly for the improved utilization of ingredients and by‐products generated on a large scale (Lemes et al. 2021).

The by‐products generated during food processing are full of nutrients and bioactive ingredients, which can be explored and offer a raw material with high added value (Almoumen et al. 2024). Global food waste is already approaching 1.4 million tons and is projected to reach 2.6 million by 2025 (Sinha and Tripathi 2021). A large part of the plant by‐products generated in this process could be used to produce ingredients with high nutritional and functional value (Lemes et al. 2021).

A general classification of plant by‐product resources from which dietary fibers can be extracted is helpful to demonstrate their diversity and industrial potential. These sources can be broadly categorized as follows: (1) cereal husks, such as wheat bran, and rice and corn husks, which are rich in insoluble fibers like cellulose and lignin; (2) fruit peels and pomace, such as apple peel, orange bagasse, and mango peel, which contain soluble fibers like pectin; (3) plant processing residues, including stalks, peels, and leaves of vegetables like potatoes, carrots, and broccoli, providing a mix of soluble and insoluble fibers; and (4) oilseed residues, such as soy cake from soy or peanut skins, which are fiber‐rich and often underutilized (Hussain et al. 2022; Iqbal et al. 2022; Plakantonaki et al. 2023). This categorization clarifies the wide range of raw fiber extraction and valorization materials.

Recent market trends indicate a growing interest in using plant‐based fibers, driven by increased consumer demand for functional foods, clean‐label products, and sustainability (Prasad et al. 2025). Innovations in fiber manufacturing and characterization include non‐thermal extraction methods (e.g., ultrasound o high‐pressure processing), enzymatic treatments to enhance functional properties, and the application of nanotechnology to produce nanofibers with improved bioactivity and textural performance. These technological advances enhance extraction efficiency and expand the applicability of fibers across various sectors, including food, pharmaceuticals, and biodegradable packaging. Such developments underscore the industrial and economic relevance of plant‐derived fibers in a circular bioeconomy (Iqbal et al. 2022).

Another way that positively influences the idea of using plant by‐products generated by the agroindustry is the implementation of the global sustainable development program offered by the World Health Organization (WHO), linked to the Zero Hunger program. Through agriculture, such programs give participating countries the responsibility of finding ways to substantially reduce waste and thus distribute food to the 9 billion people who are expected to inhabit the planet by 2050 (Callegari and Stoknes 2023). Furthermore, the valorization and possible internationalization of local foods are encouraged for these programs (Blesh et al. 2019).

## 3 Plant Agro‐Industrial By‐Products as Potential Raw Materials to Obtain Food Ingredients

**FIGURE 2:**
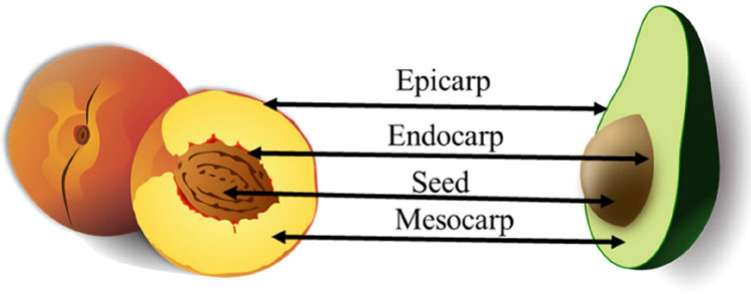
The botanical division of edible fruits into pericarp (mesocarp, epicarp, and endocarp) and seed.

Processing products of plant origin usually uses the edible part as raw material, often the pulp or seed. Figure 2 shows the botanical division of the fruits, highlighting their parts.

The pericarp originates from the ovary wall and can be divided into three parts: epicarp, mesocarp, and endocarp. The epicarp is the external covering formed by the external epidermal tissue to protect the fruit. The mesocarp, also popularly known as pulp, corresponds to the intermediate and most developed layer, mainly made up of fleshy fruits. The endocarp is the innermost layer and is generally poorly developed, formed by parenchymatic or sclerenchymatic tissues (de Souza 2022). The vast majority of the epicarp and endocarp constitute the agro‐industrial by‐product fraction, some of which have the presence of seeds that are discarded according to the desired process (Freire et al. 2023).

Generally, the raw material used in the food industry consists of pulp (mesocarp), which can include the peel (epicarp) and, in some cases, the seed. Therefore, the other plant parts become by‐products (Gómez‐García et al. 2021; Mateos‐Aparicio 2021). By‐products from epicarp or peel represent around 20%–30% of the 270 million tons produced worldwide, followed by 40% coming from mesocarp (mainly bagasse) after food production, and 30% from endocarp, which are discarded (Mallek‐Ayadi et al. 2018). In chemical composition, the by‐products generated include a large amount of fiber (such as pectin and cellulose), protein, vitamins, and antioxidant ingredients (Jamwal et al. 2023). Compared to synthetic fibers, plant fibers are renewable resources, have a 100% biodegradable life cycle, and have fewer abrasive materials with low density and high deformability. Thus, plant fiber demonstrates an excellent potential for reuse and applicability (Fayaz et al. 2022).

## 4 Plant Fiber Structure

**FIGURE 3:**
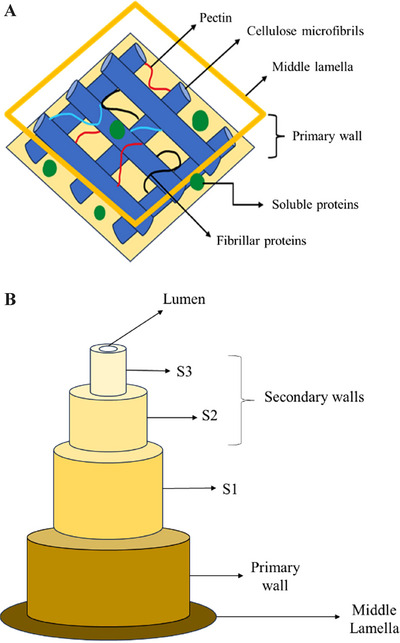
Scheme demonstrating a generic structure of plant fiber (A) and cell wall (B).

The fiber structure (Figure 3A) presents a plant cell wall composed of a mixture of polysaccharides with a predominance of cellulose, proteins, mineral salts, and phenolic ingredients, where the matrix can be more rigid or looser depending on cellular behavioral needs. Most of the components of fiber molecules are polar substances (e.g., lignin, cellulose, hemicellulose, and pectin) united by Van der Waals interactions and hydrogen bonds, which provide greater stability and malleability and make them desirable materials for various applications (de Souza 2022).

Immediately after the middle lamella (a thin protective layer), there is the primary wall, which is the outermost and most flexible layer of plant cells, where cellulose is found in the form of microfibrils (organized structures that link together to form fibrils), approximately 10–25 nm in diameter (Wohlert et al. 2022). Next to cellulose, it is possible to find hemicellulose molecules, which have structural characteristics that prevent them from forming aggregates, but they bond together through hydrogen bonds (Figure 3A) (Rao et al. 2023).

Cellulose is a homopolysaccharide with a linear and rigid structure composed of β‐D‐glucose units linked by β‐1,4 glycosidic bonds, resulting in a high molecular weight (variable according to the polymerization degree). Cellulose is a main cell wall component responsible for the plant's rigidity, support, and resistance (Chaudhary et al. 2022). In some plants, cellulose can be stored as an energy reserve, participating in cellular communication processes and regulating water absorption (Y. Kim et al. 2020). With a hygroscopic characteristic (insoluble in water and capable of absorbing it) (Fang et al. 2023), cellulose also acts as an insoluble fiber for human consumption (Chaudhary et al. 2022).

Hemicellulose is a lignocellulosic structure considered the second heterogeneous hydrophilic polysaccharide, abundant in the plant cell wall, being only surpassed by cellulose (Rao et al. 2023). As a structural polysaccharide, its chemical composition and structural characteristics may vary between plant species, subcellular compartments, and developmental stages (Dias et al. 2022). Some of the most common sugars in its molecular formula include xylan, mannan, galactan, arabinan, and glucuronic acid, which may vary according to its plant source (Xu et al. 2023). Due to its complex branched structure with β1→4 bonds, tangles of hemicellulose and cellulose through hydrogen bonds serve as a “bridge” for lignin deposition (Chaudhary et al. 2022).

Pectins are present in part of the primary walls of the fibrous structure. They are biopolymers formed by galacturonic acid and are abundant in the primary walls of some dicotyledons and monocotyledons. In the cell wall, pectin molecules are strongly linked to other polysaccharides and proteins (Milošević and Antov 2022). Conventionally, pectin extraction is performed via hot chemical methods. However, in some cases, enzymes are also applied as hydrolysis agents for other polysaccharides to release pectin (Adetunji et al. 2017). Enzymatic extraction is advantageous because it enhances pectin recovery and can yield pectic substances with diverse structural, physicochemical, and functional properties compared to chemical extractions. In addition, regardless of the method chosen, pre‐processing of the plant's raw material is recommended to facilitate pectin extraction (Zoghi et al. 2023).

The secondary wall provides structural support to the cells, contributes to the cell wall's resistance, and has sublayers S1, S2, S3, and S4 (exclusive to some plant matrices) (Figure 3B). The S1 layer (sublayer 1) is thicker than the primary wall. S1 contains additional components, such as lignin, that increase the rigidity of the cell wall and provide additional mechanical strength and protection to the cell. The beginning of the secondary layer is the S2 layer (sublayer 2), which is responsible for conferring the ability to retain water, resist enzymatic digestion, and benefit human health, primarily by promoting intestinal regularity. The S3 layer (sublayer 3) and the S4 layer (sublayer 4 located just below S3) are composed of cellulose and hemicellulose, contributing to the rigidity and resistance of the secondary wall (Koshani et al. 2025). Finally, the lumen is the internal cavity of the plant cell that contains the cytoplasm, cell organelles, and other cellular components essential for the cell's vital functions and where metabolic and storage processes occur (Mukherjee and Ghosh 2023).

Lignin is an essential component of the plant cell wall. This molecule is an amorphous three‐dimensional polymer composed of monolignols, such as coumaryl alcohol, coniferyl alcohol, and sinapyl alcohol. These molecules are linked by ether and carbon‐carbon bonds, which confer rigidity and strength to lignin (Albersheim et al. 2010). Lignin is a small complex fraction in the cell wall, responsible for mechanical support, waterproofing, resistance to decomposition, water transport, and carbon storage (Ali et al. 2024).

The choice of lignin extraction method (each with its advantages and disadvantages) varies according to the type of biomass, the purity of the desired lignin, and its intended final applications (Nuamduang et al. 2024). Lignin can be extracted by (i) acid hydrolysis, which hydrolyzes cellulose and hemicellulose, releasing lignin as solid residue. (ii) Kraft process involves applying NaOH and Na_2_S to break the bonds between cellulose and dissolve the lignin. In addition, (iii) a specific thermal process for wood, where the lignin sample dissolves in the aqueous solution of HSO_3_^−^ known as the sulfite process, can also be applied (Tanis et al. 2023).

Furthermore, the cell wall of some plants may contain mucilage, which is another polysaccharide composed of L‐arabinose, D‐galactose, and galacturonic acids, having soluble and insoluble fractions (Dybka‐Stępień et al. 2021; Pontes et al. 2020). Gums are also found in long‐chain hydrocolloids that are dispersible in aqueous medium and insoluble in organic solvents (Jayakody et al. 2023). These components can be quantified together with fibers and constitute food production ingredients.

In summary, the polysaccharides present in dietary fibers play a fundamental role in determining their functional properties. These compounds influence not only the interaction of fibers with the intestinal environment—such as lipid digestibility and fermentation—but also directly affect their technological applications in food formulation. Therefore, understanding the composition and behavior of these polysaccharides is essential to optimize the use of dietary fibers as functional and health‐promoting ingredients.

## 5 Plant Fiber can be a Dietary Fiber

The possibility of using food processing by‐products as raw material for producing ingredients appears to be a strategy to reduce waste and generate income indirectly. This process requires preparation, extraction, and/or fibrous modification steps, which can produce ingredients with varying characteristics that can be applied in different food products (Lemes et al. 2022; Lemes et al. 2021).

Crude fiber is an analytical term used when determining the indigestible portion of plants, that is, the ingredient of plant substances that are resistant to extraction by acids and alkalis (Saura‐Calixto et al. 2000). Meanwhile, dietary fiber consists of polymers of indigestible polysaccharides and lignin and can be soluble and insoluble (Williams et al. 2019).

Soluble dietary fibers (SDF) (e.g., pectin, β‐glucan, fructooligosaccharides, galactooligosaccharides, and inulin), which are important from a physiological and functional point of view, are defined according to their solubility characteristics. Soluble dietary fiber has a morphology that allows it to be dissolved and fermented by intestinal bacteria, producing short‐chain fatty acids (SCFAs) (e.g., butyrate) that provide energy to the colon cells (approximately 2 kcal/g). However, it is important to note that the exact amount may vary depending on the specific type of soluble fiber (Guan et al. 2021).

Insoluble dietary fibers (IDF) remain solid during their passage through the gastrointestinal tract. However, emerging evidence suggests that IDF can be fermented by specific intestinal bacteria, generating metabolites that are beneficial to intestinal health (e.g., short‐chain fatty acids) (Baky et al. 2024). IDF can retain water, thus promoting an increase in fecal volume, stimulating the contraction of intestinal muscles, and preventing constipation (P. Zhou et al. 2024). In addition, IDF promotes a mechanical function in the digestive tract, influencing the process of lipid digestion through the absorption of bile salts (Tang et al. 2022; Tornberg 2023).

The composition of the different dietary fiber sources can impact their technological properties. Therefore, the combination of different dietary fiber sources can also be used to develop products specifically tailored to individual needs (Dybka‐Stępień et al. 2021; Sabater et al. 2022). Additionally, the choice of extraction or processing method for the by‐product to produce fibrous ingredients is also essential, as it affects its characteristics and applicability (Atencio et al. 2021).

## 6 Processing of Fibrous Ingredients From Plant Agro‐Industrial By‐Products

As previously stated, agro‐industrial food by‐products present considerable levels of macronutrients such as carbohydrates including fibers, proteins, and some micronutrients such as carotenoids and peptides, among others (Coman et al. 2020; Gonçalves et al. 2023). Using by‐products is advantageous, particularly for individuals with special dietary needs, such as those with celiac disease, as adapting their diet can be challenging (Egea et al. 2023).

**FIGURE 4:**
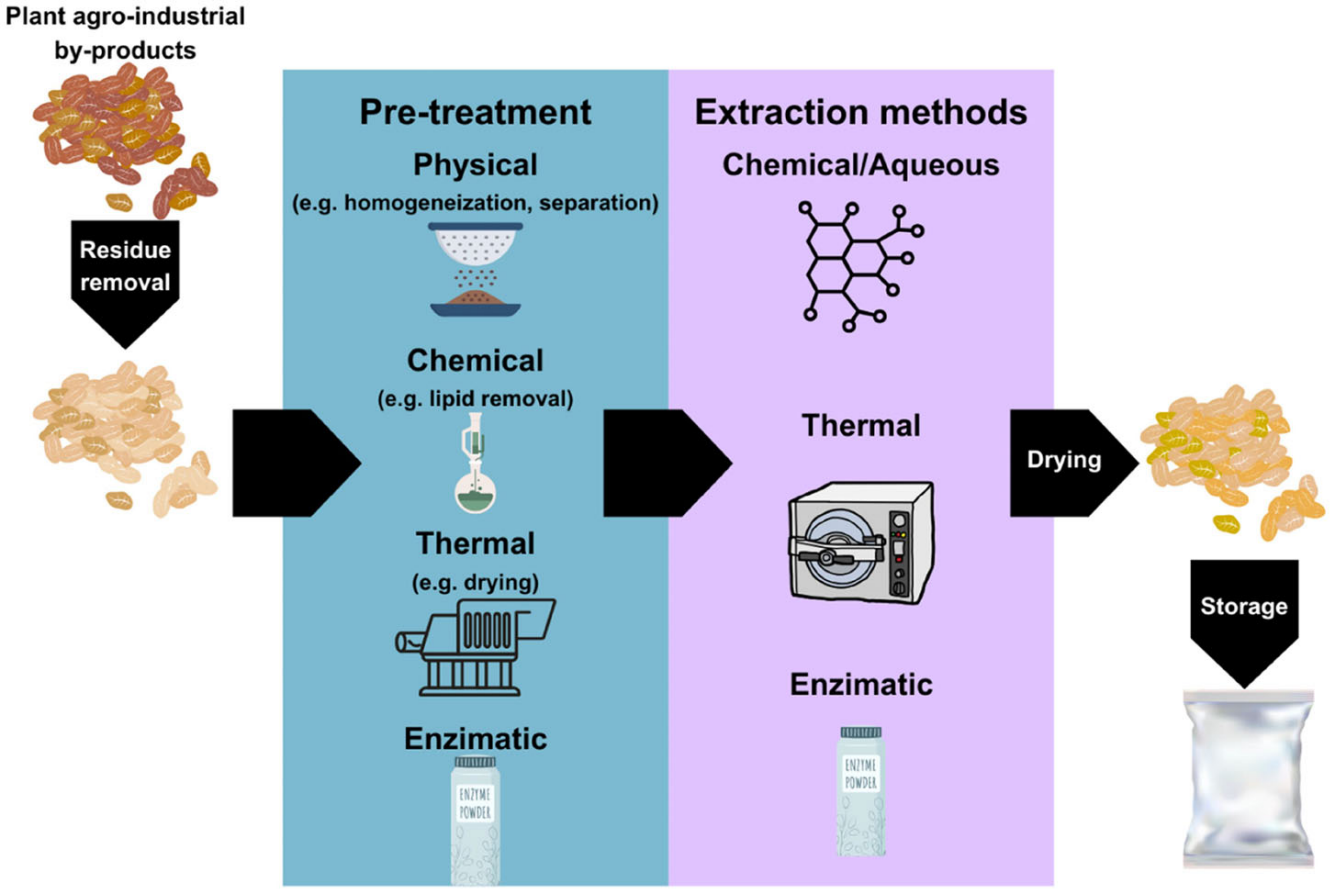
Stages of production of fibrous ingredients from agro‐industrial plant by‐products.

Extracting and isolating ingredients present in generated by‐products becomes a utilization strategy that can reduce waste generation and its negative impact on the environment, and has been increasingly used. To this end, it is necessary to adopt processes that utilize by‐products more effectively and enhance the extraction and production of ingredients (Lemes et al. 2022). Several steps are necessary to achieve this, including residue removal, pre‐treatment (such as grinding for homogenization, particle size separation, and extraction of residual lipid contents), extraction, and material storage (Figure 4).

### 6.1 Pre‐Treatment of the Agro‐Industrial By‐Product to Obtain the Fibrous Ingredient

The processing conditions to which the plant material is subjected can alter its composition and microstructure, thereby aiding subsequent processing. Therefore, maintaining standardization in the fibrous sample preparation stage is crucial, as different extraction methods can yield varying results, including variations in chemical composition, structure, and functional properties (Wang et al. 2022).

In this sense, sample preparation is a fundamental step. Generally, samples are subjected to a preliminary drying pre‐treatment (40°C –60°C) and are subsequently crushed to homogenize the particle size and increase the contact surface in the other stages of the process. Particle size is important because it impacts the functional properties of the ingredients (Tornberg 2023), and a reduction in particle size can affect the surface structure of the fiber, leading to physical‐chemical and functional changes, in addition to the possibility of reduced water absorption (Mohammed et al. 2022).

Furthermore, the removal of lipid content has also been used as a pre‐treatment as it can demonstrate benefits in later stages, mainly by increasing physical availability in the extraction stage (Wang et al. 2022). Thus, any cold or hot degreasing step can be used, or even more specific steps, such as immersing the sample by‐product in boiling 96% (v/v) ethanol and washing with 70% ethanol. In the latter case, Zhang et al. (2017) reported that low‐molecular‐weight sugars, organic acids, and inorganic salts are also removed, and that enzymes are inactivated. Biological methods, such as enzyme application, can also be used as pre‐treatments to remove other carbohydrates and proteins (Lemes et al. 2021; Liu et al. 2021).

### 6.2 Generic Process for Extracting Fibrous Ingredients From By‐Products

**FIGURE 5:**
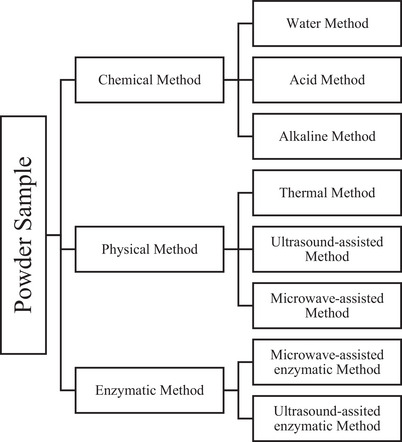
Diagram of different methods of fiber extraction from agro‐industrial by‐products.

Fiber extraction is based on the type of ingredient desired (such as soluble or insoluble). This extraction can be chemical, enzymatic, enzymatic‐chemical, physical, or a combination of more than one of these methods (Ma and Mu 2016). Figure 5 shows a diagram of the different fiber extraction methods from agro‐industrial by‐products after carrying out the steps described in item 6.1.

Table 1 shows the studies in the literature in the last 5 years that used some extraction methods to modify plant by‐products.

**TABLE 1:** Ingredients or materials were obtained by applying distinct extraction methods and modifications from plant by‐products, where ↑ indicates an increase, ↓ indicates a decrease, and = indicates no change.

Ingredient/material obtained	Modified fraction	Applied method	Reagent	Main results	Reference
Coffee parchment	SDF and IDF	Enzymatic and aqueous	Simulated digestive enzymes and water	Both methods: ↑ IDF and lignin contents; OHC and LGC; and GAC and α‐amylase inhibition. ↓ Cellulose content and starch digestibility (46%). = yield (89% enzymatic and 86% aqueous). All the results indicate that the extraction of IDF does not require enzymes.	(Benitez et al. 2019)
Ginseng residue	TDF and IDF	Chemical, chemical‐enzymatic, enzymatic, ultrasonic‐assisted chemical, and microwave‐assisted chemical	Hydrogen peroxide and cellulase	Chemical‐enzymatic: ↑ SDF content (6.40 g). ↓ TDF content; GAC; α‐amylase inhibition (2.77%); and pancreatic lipase inhibition (22.61%). Enzymatic method: ↑ WHC; CEC; GAC; and PLI. Chemical‐ultrasound: ↑ OHC and α‐amylase inhibition. Microwave: ↑ WSC and GAC.	(Jiang et al. 2020)
Forced roots of Belgian endive	TDF	Aqueous	Water	↑ TDF content, WHC, and WSC (fiber concentrate). ↓ Sugar content, PA, and SL (powder sample).	(Twarogowska et al. 2020)
Bamboo shoot fiber	TDF	Chemical, enzymatic, ultrasonic‐assisted enzymatic, and shear homogeneous‐assisted enzymatic	NaOH and α‐amilase	Chemical: ↓ Protein and crystallinity index. Enzymatic: ↓ OHC and protein. Enzymatic‐ultrasonic: ↑ OHC and GAC. Enzymatic‐shear: ↑ SDF content (17.89%); WHC (8.81 g/g); and inhibition of α‐amylase activity (19.89%). ↓ Particle size (351.33 µm)	(Tang et al. 2022)
Millet bran fiber	Hydroxyl groups and TDF	Enzymatic and chemical	Cellulase, propylene oxide, and acetic anhydride	Enzymatic: ↑ IDF content (852.4 g/kg); and PC content (647.95 mg/kg); WHC (4.74 kg/kg); and WSC (0.84 L/kg). Chemical: ↑ α‐Amylase activity inhibition (34.53%) and OHC.	(Zheng et al. 2022)
Soluble and insoluble fibrous ingredient of fruits of *Rubus chingii Hu*	SDF and IDF	Aqueous, chemical, and enzymatic	Water, NaOH, HCl, and α‐Amylase	Aqueous: ↑ OHC; thermal stability; and viscosity. Aqueous and acidic SDF with more complete structure. Chemical: ↑ SDF yield (17.77 g/100 g). Enzymatic: ↑ Protein content (3.95%)	(Wang et al. 2022)
Citrus peels	IDF	Enzymatic	Pancreatic lipase	↑ AC and PLI.	(Yu et al. 2023)
Oat	IDF	Chemical	NaOH, KOH, and methanol	↑ Solubility; OAC; WAC; thermal stability; and brown hue. ↓ L* after modifications	(Kanwar et al. 2023)
Sorghum residue	TDF	Thermo‐chemical (alkaline) and thermo‐enzymatic	NaOH, HCl, and Laccase enzyme	Thermo‐Enzymatic: ↑ Thermal properties and mechanical properties. Alkaline: ↑ Tensile strength (28.57 MPa)	(Moumakwa et al. 2023)
Date by‐product	IDF	Aqueous	Deionized water	↑ Yield of functional polysaccharides; WHC; and OHC. ↓ Stability	(Almoumen et al. 2024)
Ponkan by‐products	SDF and pectin	Ultrasound and microwave assisted aqueous	Water	↑ TDF, phenolic, and flavonoid contents Optimization of the recovery of bioactive compounds.	(Chang et al. 2024)
Hordeum vulgare bark	TDF and soluble xylan	Chemical‐enzymatic	HCl, NaOH, glucoamilase, and α‐amilase	↑ Antioxidant action; thermal resistance (handling peak 266°C); SDF with prebiotic potential; and isolated xylan yield (40%)	(Chelliah et al. 2024)
Passion fruit seed	TDF	Chemist and ultrasound assisted chemist	NaOH	Chemical: ↑ TDF content (52.8%). Chemical‐ultrasound: ↑ WHC; OHC; GAC; and EC (compared to chemical method)	(Chutia et al. 2024)
Flaxseed meal	TDF	Chemical, enzymatic, and physical	NaOH, cellulase, and acrylate grafting	Chemical and physical: ↑ HC. Enzymatic and physical: ↑ Adsorption capacities to lipids, cholesterol, and nitrite ions; GAC; GDRI; and AIA.	(Huo et al. 2024)
Fibrous material from Luffa and corn husk	TDF	Chemical	NaOH	↑ WAC; structural surface; and mechanical properties.	(Islam, Supto, Rafi et al. 2024)
Grain bran	TDF	Enzymatic	Papain and α‐amylase	↑ TDF content (83.6%); preventive effect on ulcerative colitis; and metabolic regulation.	(Li et al. 2024)
Passion fruit and tamarillo residues	TDF and SDF	Chemical and microwave assisted chemical	Tartaric acid	Chemical: ↑ Pectin quality and similarity to commercial pectin. Chemical‐microwave: ↑ Pectin yield and degree of esterification	(Manjula et al. 2024)
Soy sauce residue	TDF	Enzymatic	Transaminase	↑ Hepatic glucose metabolism; antidiabetic effect; TDF content; and abundance of *Dubosiella*, *Butyricimonas*, and *Lachnospiraceae* in mice microbiota. Structural modification of fiber.	(Mo et al. 2024)
Defatted date seed powder	TDF	Ultrasound‐assisted aqueous	Water	↑ Physical properties of dough and shelf life. ↓ Creep deformation of dough.	(Ranasinghe et al. 2024)
Rice bran	TDF	Chemical‐Enzymatic	NaOH and Cellulase	↑ Enzymatic extraction yield (2% SDF and 59.5% IDF contents).	(Shaikh et al. 2024)
Pectin residue derived from mango peel	SDF and pectin	Microwave assisted chemistry	Water and citric acid	↓ In vitro digestive factors; L* (58.04); a* (12.80); b* (23.50); 6.81% moisture content; and 78.63% solubility. Modification and structural improvement of pectin.	(Srikamwang et al. 2024)
Pomegranate peel	SDF	Enzymatic and chemical	Papain, saccharifying enzyme, α‐amylase; and NaOH	Enzymatic: ↑ Resistant starch content (0.5%). ↓ Potato starch digestibility (SPS) and gel strength and hardness. Chemical: ↑ Acceleration of starch regression. ↓ Potato starch digestibility (SPS).	(Xiong et al. 2024)
Fibrous residue of *Hericium erinaceus*	SDF	Enzymatic and ultrasound‐assisted enzymatic	α‐amylase, protease, and ethanol	Enzymatic: ↑ Sodium glycocholate binding. Enzymatic‐ultrasound: ↑ Extraction; AC; bile salt binding; and pancreatic lipase inhibition by SDF content.	(Yu et al. 2024)
Glutinous rice starch and celery fibers	Mixture of starch and SDF	Aqueous	Water	↑ Thermal stability; complexation index (69.41%); resistant starch (8.15%–8.95%), contributing to the probiotic effect; thermal stability; and inhibition of starch retrogradation. ↓ Viscosity and digestibility of glutinous rice starch.	(Yu et al. 2024)
Cucumber by‐product	TDF and SDF	Enzymatic; high‐pressure assisted enzymatic; and high‐pressure microfluidization assisted enzymatic	α‐amylase, protease, and glycolytic enzymes	All methods: modified SDF content and ↑ thermal stability Enzymatic: ↑ SDF content (4.08%). Enzymatic‐High Pressure: ↑ SDF content (4.08%) and modification of insoluble fiber to soluble. Enzymatic‐microfluidization: ↑ HC and thermal stability. ↓ crystallinity and GAC.	(Zhang et al. 2024)

AC = adsorption of cholesterol; AIA = α‐amylase inhibition activity; CEC = cation exchange capacity; EC = emulsifying capacity; GAC = glucose adsorption capacity; GDRI = glucose dialysis retardation index; HC = hydration capacity; IDF = insolube dietary fiber; LGC  =  least gelation capacity; OAC = absorption oil capacity; OHC = oil holding capacity; SDF = soluble dietary fiber; TDF = total dietary fiber; WHC = water holding capacity; PA = phenolic acids; SL = sesquiterpene lactones; PLI = pancreatic lipase inhibition; WAC = water absorption capacity; WSC = water swelling capacity.

#### 6.2.1 Chemical Extraction

Chemical extraction is based on the use of (i) acidic, (ii) aqueous, and (iii) alkaline methods, with (i) and (iii) being the most widely applied. Table 1 presents ingredients or materials obtained by applying different extraction methods and modifications from plant by‐products. According to this table, chemical extraction is the most widely used method for fiber extraction due to its high extraction yield. For example, in the extraction of *Rubus chingii* Hu residues, the chemical method yielded 17.77 g/100 g of soluble fiber, which is higher than the yields of the aqueous and enzymatic extractions (Wang et al. 2022).

The (i) acidic method involves the application of acidic solutions (sulfuric acid [H_2_SO_4_] or hydrochloric acid [HCl]) in different concentrations to solubilize the non‐fibrous components of the plant matrix, allowing the recovery of fibers (Smole et al. 2019). The disadvantage of this method is that the most studied acids have toxicity that limits the extraction of fibrous ingredients for food purposes (Zheng et al. 2024). HCl was predominant in chemical extraction using acids (Chelliah et al. 2024; Moumakwa et al. 2023; Wang et al. 2022) (Table 1).

The (ii) aqueous method utilizes water and elevated temperatures, ranging from 40°C to 90°C. This method is considered green due to its low environmental impact (Twarogowska et al. 2020). In this fiber extraction method, the techno‐functional properties are increased (Table 1) such as those related to water absorption for example water holding capacity (WHC) (Almoumen et al. 2024; Twarogowska et al. 2020), water swelling capacity (Twarogowska et al. 2020), and  least gelation capacity (LGC) (Benitez et al. 2019); oil absorption (Almoumen et al. 2024; Benitez et al. 2019; Wang et al. 2022). However, the viscosity was increased when the aqueous method was applied in SDF and IDF ingredients of fruits of *Rubus chingii Hu* (Wang et al. 2022) and decreased when the method was applied in pomegranate peel (Yu et al. 2024).

The improvement of the techno‐functional properties of the fibers after the application of the aqueous method can be due to a combination of structural and compositional factors, such as the removal of soluble components such as sugars, phenolic compounds, and sesquiterpene lactones. These compounds increase the porosity of the capillary structure of the fibers (Twarogowska et al. 2020) and the insoluble fraction of the fiber by inducing the formation of insoluble complexes between polysaccharides and proteins or phenols present in the cell wall (Liu et al. 2020). In addition, these compounds alter the morphology and structure of the fibers, increasing the contact surface and creating active sites for the absorption of water and oil (Almoumen et al. 2024).

In (iii) alkaline extraction, sodium hydroxide (NaOH) solution is generally used due to its greater action in food matrices and ease of neutralization. The action of NaOH is based on the rupture of intercellular adhesive forces, which consequently allows NaOH to access groups of the cell wall structure, forming covalent bonds. This process results in more completely separated fibers and greater roughness of the fibrous surface (Barreto et al. 2011). To ensure greater effectiveness in alkaline treatment, conditions such as concentration, time, and temperature of the solution must be previously studied. In general, the concentration varies from 1% to 10% (Wang et al. 2022), with a time of 12–48 h (MMa and Mu 2016; Wang et al. 2022), and a temperature of 4°C–120°C (Manjula et al. 2024; Moumakwa et al. 2023) (Table 1).

The improvement of the techno‐functional properties of the ingredient after the application of alkaline extraction is the solubilization of hemicellulose and lignin, leaving the cellulose exposed (Moumakwa et al. 2023); increased porosity and surface area of the fibers; exposure of free hydroxyl groups that favor interaction with water and oil (Wang et al. 2022); and improved compatibility with hydrophobic matrices such as polymers in composites (Moumakwa et al. 2023).

Other chemical reagents are being tested to improve the characteristics of the fibers obtained. Huo et al. (2024) used acrylate‐grafting as a chemical reagent to promote chemical reactions and modify and improve the properties of flaxseed cake fibers. The authors compared this method with the chemical method using sodium hydroxide (NaOH) and enzymes (cellulase). They found increased techno‐functional (WHC) and chemical (cation exchange capacity) properties.

#### 6.2.2 Physical Extraction

Physical, mechanical, or thermal extractions are considered when the desired fibers make up 60%–80% of the desired material, making them easier to obtain (Amel et al. 2013). Thermal extraction can be combined with alkaline or aqueous chemical methods, utilizing the time–temperature relationship under conditions of high pressure and temperature, typically achieved using an autoclave (Huo et al. 2024; Kanwar et al. 2023; Twarogowska et al. 2020; Wang et al. 2022).

As shown in Table 1, studies involving physical extraction are scarce. However, Huo et al. (2024) obtained satisfactory results, including increased water retention capacity and enhanced swelling in water, which may be attributed to the greater exposure of hydroxyl groups and the creation of a more porous structure that facilitates water interaction. This more porous and open structure can also result in greater fluidity, that is, lower viscosity.

#### 6.2.3 Enzymatic Extraction

Among the biological methods, enzymatic extractions are well‐established and, when compared to chemical methods, are more advantageous due to their lower corrosion potential, as they utilize a lower pH, a lower content of degraded compounds in the sample, and a higher yield (Lemes et al. 2021; Tang et al. 2022). On the other hand, using enzymes requires process control and, therefore, becomes a more expensive method. The choice of enzymes varies according to the by‐product used and the objective and desired characteristics for the fibrous ingredient developed. The most commonly used enzymes are α‐amylases, proteases, amyloglucosidases, and cellulases (Ramesh et al. 2020).

Mixed or assisted methods, such as those involving microwaves, ultrasound, and shear, are techniques that, in association with enzymatic methods, break the plant structure more efficiently and increase the solvent's accessibility to the internal structure, thereby enhancing extraction efficiency (Jiang et al. 2020). These methods have long extraction times, require sensitive enzymes, and strict pH and temperature conditions, especially on a large scale (Tornberg 2023) and with a wide variation in the structural properties of the fibrous ingredient (Wang et al. 2022).

Table 1 shows that the enzymes most commonly used to modify fibers from plant by‐products are α‐amylase (Chelliah et al. 2024; Karthik and Kalyani 2021; Li et al. 2024; Tang et al. 2022; Wang et al. 2022; Xiong et al. 2024; Yu et al. 2024; Zhang et al. 2024) and cellulase (Huo et al. 2024; Jiang et al. 2020; Shaikh et al. 2024; Zheng et al. 2022). However, some other enzymes, such as simulated digestive (Benitez et al. 2019), saccharifier (Xiong et al. 2024), protease (Yu et al. 2024; Zhang et al. 2024), and glycolytic enzymes (Zhang et al. 2024), are being used aiming at greater extraction efficiency and better characteristics of the extracted and modified fibers.

Although extraction methods can directly impact the yield and quality of the fiber obtained, in the case of coffee parchment, both the enzymatic and aqueous methods presented high yields (89% and 86% for the enzymatic and aqueous methods, respectively) (Benitez et al. 2019). In addition to the similarity in yield, both increased the content of IDF, lignin, oil holding capacity (OHC), and gel forming capacity, in addition to inhibition of α‐amylase and a reduction in starch digestibility by 46% (Table 1).

For ginseng residue, the chemical‐enzymatic method increased the SDF content to 6.40 g. Still, it reduced the total fiber amount, glucose adsorption capacity, and the inhibition of α‐amylase (2.77%) and pancreatic lipase (22.61%) enzymes (Table 1). On the other hand, the enzymatic‐only method increased water‐holding capacity (WHC), cation exchange capacity, glucose adsorption capacity, and pancreatic lipase inhibition, indicating improved functionality. However, the exact yield values were not reported (Jiang et al. 2020).

On the other hand, the extraction of millet bran fiber using enzymes resulted in a high content of IDF (852.4 g/kg) and phenolic compounds (647.95 mg/kg), in addition to WHC of 4.74 kg/kg and water swelling capacity of 0.84 L/kg. The chemical method stood out for its greater inhibition of α‐amylase activity (34.53%) and oil retention capacity.

#### 6.2.4 Other Methods

The combination of different modification techniques (chemical, physical, and/or enzymatic) is capable of disrupting the structure of fibrous materials and enhancing their porosity, thereby exposing specific groups within their structure, leading to variations in the physicochemical characteristics of dietary fiber (Kanwar et al. 2023). On the other hand, they can also increase the cost of production; therefore, they must be studied and compared with the techniques in isolation.

The combination of conventional methods (chemical and enzymatic) with increased temperature, ultrasound, microwaves, homogeneous shear, high pressure, and microfluidization offers several advantages, including gentle operating conditions, straightforward scale‐up, high yields, and improvement in the physicochemical and techno‐functional characteristics of the fibers (Table 1). For example, combined methods, such as chemical‐ultrasonic and microwave, promoted additional increases in OHC, glucose adsorption capacity, and solubility in ginseng residue (Jiang et al. 2020) (Table 1).

In the case of passion fruit seed residue, although simple chemical extraction promoted a 52.8% increase in total dietary fiber content, the ultrasound combination improved functional properties, such as water holding capacity (WHC), oil holding capacity (OHC), glucose adsorption capacity, and emulsifying capacity. However, quantitative values were not specified in this comparison (Chutia et al. 2024).

Another method, high‐pressure microfluidization combined with enzymatic extraction, was applied to the cucumber by‐product, resulting in increased thermal stability and hydration capacity, as well as the modification of IDF into SDF (Zhang et al. 2024).

Table 2 summarizes the main methods discussed in this review. This table highlights the advantages, limitations, and optimal operational conditions of each strategy, providing a more straightforward overview of the trade‐offs associated with different fiber extraction and modification approaches. This comparative synthesis aims to assist researchers and industry professionals in selecting the most appropriate approach based on the desired characteristics of the fiber ingredient and its intended application.

**TABLE 2:** Comparison of main extraction and modification methods for plant fibers from agro‐industrial by‐products.

Method	Advantages	Limitations	Ideal conditions	References
Chemical (acid, alkaline, aqueous)	High extraction efficiency effective lignin and hemicellulose removal; and improves functional properties	Use of toxic reagents (strong acids); potential for harmful residues; requires neutralization	Acid: HCl 1–2 M, 60°C–90°C, 1–2 h; Alkaline: NaOH 1%–10%, 4°C–120°C, 12–48 h; Aqueous: 40°C–90°C, 1–4 h	(Chelliah et al. 2024; Huo et al. 2024; Wang et al. 2022)
Enzymatic	High selectivity; less degradation; and maintains fiber integrity	High cost; sensitive to pH and temperature; and longer processing time	Enzymes such as α‐amylase, cellulase, protease; pH 4.5–7.0; 35°C–55°C; 1–24 h	(Jiang et al. 2020; Tang et al. 2022; Yu et al. 2024)
Physical (thermal, mechanical)	Simple process; low cost; can increase porosity; and accessibility	Low selectivity and lower yield when used alone	Autoclaving: 121°C for 15–30 min; milling; pre‐treatment with microwave or ultrasound	(Amel et al. 2013; Huo et al. 2024)
Combined methods (physical‐chemical, enzyme‐assisted)	Enhances techno‐functional properties; synergistic effects; and higher yields	Higher cost and requires precise parameter control	Ultrasound + enzymatic: 20–40 kHz, 50°C–60°C, pH 5–6; chemical + thermal: 90°C–121°C with NaOH or HCl	(Chutia et al. 2024; Jiang et al. 2020; Shaikh et al. 2024)

**HCl**: hydrochloric acid; **NaOH**: sodium hydroxide.

### 6.3 Industrial Feasibility and Scale‐Up Considerations

Although various fiber extraction methods (Table 2) have been optimized at the laboratory level, their industrial scalability remains a key consideration for their application. Aqueous and alkaline extractions are among the most viable options for scale‐up due to their simplicity, reagent availability, and lower energy demands, which contribute to more favorable production costs. In contrast, despite yielding fibers with superior functionality, enzymatic and combined techniques may face economic and operational limitations due to higher costs, complex control requirements, and longer processing times. Emerging technologies such as ultrasound‐ and microwave‐assisted extractions show promise but require further optimization for industrial adoption. Therefore, both technical performance and economic feasibility should guide the selection of extraction methods for commercial applications (Kanwar et al. 2023; Shaikh et al. 2024; Wang et al. 2022).

Using ingredients obtained by extracting plant fibers in food is subject to strict regulatory requirements, which vary by jurisdiction. In general, these ingredients must be recognized as safe for human consumption. They are often classified as “novel foods” from sources not traditionally consumed or undergo innovative extraction and modification processes. The European Union, through Regulation (EU) 2015/2283 (EFSA 2016), requires a prior safety assessment by the European Food Safety Authority (EFSA). In contrast, in the United States, GRAS (generally recognized as safe) status must be demonstrated to the Food and Drug Administration (FDA).

Additionally, it is essential to verify the absence of chemical, microbiological, and allergenic contaminants, as well as demonstrate the ingredient's stability and technological functionality in various food matrices. Compliance with these requirements necessitates the standardization of extraction methods, detailed physical‐chemical characterization, and toxicological validation, thereby ensuring safety, transparency, and traceability throughout the production chain. Therefore, aligning with international regulatory guidelines becomes essential for commercializing these ingredients in processed foods.

## 7 Technological Properties of Fibrous Ingredients

Fibers can present technological properties (WHC, foaming capacity, and the intrinsic expansion force of the fiber) and functional properties (beneficial effects on humans) that are directly related to the degree of degradation and/or modification of the by‐product and the physiological function (Beikzadeh et al. 2020).

Technological properties are related to the behavior of each ingredient in a food system and become an essential study before food application. Investigating the composition and microstructure of the fibrous ingredient can be a key factor in determining its technological properties. Once modified by some processes described in item 6.2, technological properties can become greater or different depending on the type and characteristics of the process, such as hydration and oil retention (He et al. 2023).

The technological properties of the fibrous ingredient depend on factors such as the ratio between SDF and IDF contents, particle size, conditions used in the extraction process, the structure of the plant polysaccharide, and plant source (Pathania and Kaur 2022). The technological properties of the insoluble fiber fraction depend directly on factors such as the amount of water‐insoluble solids, the area of large particles, and the hardness of these particles. In contrast, the soluble fiber fraction includes its physical structure as one of the most critical factors (Tornberg 2023).

Since applying the fibrous ingredient affects the technological characteristics, such as color, flavor, and especially the texture of the final food product, knowledge of these properties guides its application (Twarogowska et al. 2020). The technological properties are divided into hydration properties, hydrodynamic properties, and surfactants (Murray 2020), and will be described in the following subsections.

### 7.1 Hydration Properties

Hydration properties are the ability of a molecule to bind to water, including WHC, OHC, emulsifying capacity, foaming capacity, and others. Structural differences in terms of the molar mass of the fibrous chain directly influence the ability of molecules to bind and absorb water molecules (Savlak et al. 2016).

Particle size is directly related to the hydration speed of the fibrous molecule. The hydration layers contained within the molecule are linked by hydrogen bonds and dipole–dipole interactions or physically retained in microcapillaries (Ma and Mu 2016).

Also, the hydration property is greatly affected by the SDF content present in the fibrous ingredient (Gill et al. 2021), which occurs because SDF have a greater capacity to retain water, thereby improving the hydration layers within the molecule and facilitating their swelling and gelation (Zhang et al. 2017).

The correct assessment of hydration properties is critical because it indicates the type of food in which the ingredient can be used. Higher hydration suggests a good potential for the fibrous ingredient to reduce calories, increase volume, improve texture, provide a firmer structure, or impart softness, depending on the type of fiber and its interaction with other components, thereby improving the stability of the final food products (Benitez et al. 2019). Some fibers can retain water, as demonstrated above, which can be helpful in food products to maintain moisture, extend the product's shelf life, and improve sensory quality (Kapoor et al. 2016).

In certain foods, fiber can contribute to physical stability, helping to prevent phase separation or sedimentation of solid particles. The texture contribution may also occur because some soluble fibers exhibit peculiarities, including loose structures with high porosity and low crystallinity, which allows them to expose more polar groups and binding sites (Xiong et al. 2022).

In addition, greater hydration increases the dilation of dietary fiber. With this behavior, dilated fiber promotes satiety, supports weight control and appetite regulation, enhances intestinal volume, and prolongs the absorption time of other food components (Liu et al. 2021; Martínez et al. 2012). The evaluation of dietary fiber expansion can be done by physical (e.g., swelling index, fiber density, fiber porosity, specific surface area, etc.), chemical (e.g., fiber solubility and viscosity of the fibrous solution), enzymatic (e.g., in vitro digestion and fiber permeability), and microscopic (Lastra Ripoll et al. 2021) methods.

In general, the properties of WHC, hydration capacity (HC), water swelling capacity (WSC), and water absorption capacity (WAC) increase when the different modification methods are applied to plant by‐products, as can be seen in Table 1. Applying these modified fibers with intensified moisturizing properties will depend on the proposed objective.

#### 7.1.1 Water Holding Capacity (WHC)

Water retention/absorption/swelling capacity or hydration capacity is the ability of a wet material to retain water when subjected to some external force (centrifugal, gravity, or compression), indicating the amount of water that remains bound to the hydrated fiber. The capacity to retain water is one of a fiber's most important functional properties, encompassing the sum of bound water, hydrodynamic water, and physically trapped water. Water retention and expansion capacity refer to the hydration properties of the fiber and are defined as indicators of these properties (Lan et al. 2012; Maurya et al. 2015).

Depending on the structure of the fibers analyzed, the configuration of the protein, fiber, and the protein content of the biopolymer, greater retention between hydrocolloids and water is possible (Lastra Ripoll et al. 2021). For example, hydrophilic components such as cellulose and hemicellulose can cooperate to prevent water from being adsorbed on the fiber's surface, thereby keeping it trapped within its structure, which leads to improved water retention and swelling capacities (He et al. 2023).

#### 7.1.2 Oil Holding Capacity (OHC)

Oil retention/absorption capacity is the amount of oil absorbed and retained when subjected to some external force (centrifugal gravity or compression). This property can be altered due to apparent density, surface characteristics, and the hydrophobicity of the molecules that make up the fibrous material (Cui et al. 2019).

A material's oil retention capacity is often observed when aiming to reduce serum cholesterol levels by absorbing oil or fat in the intestinal lumen. Thus, it is defined as the amount of oil retained in the fibers, which enables them to reduce serum cholesterol levels (Yang et al. 2022).

By‐products rich in dietary fiber can also be enhancers of oil retention. Like WHC, OHC is also related to surface properties, total charge density, viscosity, and fiber thickness. In addition, there is a positive relationship between the hydrophilic and hydrophobic groups of fiber particles (Wang et al. 2022).

Fiber modification methods from plant by‐products tend to increase the oil absorption capacity (OAC) and OHC properties (Table 1). However, Tang et al. (2022) obtained a reduction in OHC when they used only the enzymatic method and an increase in OHC when they associated the enzymatic method with ultrasound.

### 7.2 Surfactant Properties

Surfactants are substances that can reduce surface tension and stabilize the interface, increasing the lubricating properties of a liquid. A structure may present detergency, emulsification, lubrication, foaming capacity, solubilization, phase dispersion, and swelling capacity (Drakontis and Amin 2020). Among the surfactant properties are foaming capacity and stability, emulsifying capacity, and emulsifying stability.

#### 7.2.1 Emulsion Capacity and Stability

An emulsion is an unstable thermodynamic system, prone to flocculation, coalescence, and creaming, which can lead to phase separation and shorten the shelf life of foods (Yan et al. 2020). Thus, the emulsifying capacity is the ability of a molecule to act as an agent that facilitates the solubilization and dispersion of two immiscible liquids (Lan et al. 2012).

Emulsion stability is the ability to maintain an emulsion and its resistance to ruptures, which can present greater resistance the higher the protein content, since proteins decrease surface tension as a result of electrostatic repulsion on the surfaces of molecules (Lastra Ripoll et al. 2021).

Chutia et al. (2024) obtained an improved emulsifying capacity of a fibrous food ingredient from passion fruit seeds when it was obtained by a chemical method followed by ultrasound.

### 7.3 Hydrodynamic Properties

Hydrodynamic properties describe the behavior of fluids, and these properties play an essential role in the texture, consistency, and viscosity of foods, mainly because they are related to how water interacts with the food matrix (Diaz et al. 2004; Sittikijyothin et al. 2005). Rheological properties study how this interaction with water influences the deformation and behavior of materials when subjected to an external force (Figura and Teixeira 2007).

Dietary fiber plays a crucial role in viscosity because it can provide properties in the food system without promoting undesirable structural changes. It is directly proportional to the molecular weight and length of the fiber chain. As they increase, viscosity also tends to increase (Kim and Paik 2012; Pathania and Kaur 2022; Zhou et al. 2021). Except for mineral fibers, dietary plant fibers have an affinity for water, which influences the production of swelling directly linked to water absorption, but they do not have thermoplastic properties (Van de Velde and Kiekens 2001).

Soluble fibers have numerous functional properties, which are one of the factors that arouse interest in the modification of insoluble fibers (Sun et al. 2022). Insoluble plant fibers can adhere in groups, forming networks with a much higher modulus of elasticity than the network formed in soluble fibers (Tornberg 2023).

Adding excessive amounts of fiber to food products can compromise factors such as the formation and continuity of protein networks (specifically the gluten network), requiring evaluation of its addition (Egea et al. 2023).

To enhance the understanding of how dietary fibers influence rheological and textural parameters in food systems, Table 3 presents a comparative summary of recent studies investigating plant‐derived fibers' incorporation into model food matrices. These applications illustrate the diverse roles that fibers can play, including acting as prebiotics, fat replacers, texture enhancers, and emulsifiers. By analyzing the functional impacts reported across different food systems, this synthesis highlights how fiber structure, extraction method, and source significantly influence the technological and physiological performance of the final product.

**TABLE 3:** Applications of plant fibers in food model systems: Functional roles and technological impacts.

Fiber source	Food model	Functional role	Observed effect	Reference
Coffee parchment fiber	Bakery product	Texture modifier and dietary fiber	Increased hydration, improved texture, and water retention	(Benitez et al. 2019)
Bamboo shoot fibers	Functional beverage	Prebiotic potential	Improved viscosity and hypoglycemic activity in vitro	(Tang et al. 2022)
Barley and triticale fiber	Meat analogue	Texture modifier and gelling agent	Increased hardness and cohesiveness and influenced emulsion stability	(Xiong et al. 2022)
Camellia seed fiber (fermented)	In vitro digestion model	Prebiotic and lipid‐lowering	Lowered simulated cholesterol absorption and fermentation generated beneficial SCFAs	(Yang et al. 2022)
Citrus peel fiber (varied particle sizes)	Fat‐rich food matrix	Fat absorption regulator	Adsorbed bile salts and lipids and suggesting hypolipidemic action	(Yu et al. 2023)
Barley husk (enzymatic‐alkaline extraction)	Food hydrogel	Techno‐functional enhancement	Improved WHC and emulsification	(Chelliah et al. 2024)
Passion fruit seed fiber (alkaline + ultrasound)	Emulsion system	Emulsifier/stabilizer	Increased emulsifying capacity and stability	(Chutia et al. 2024)
Fermented soy sauce residue fiber	Diabetic model (mice)	Functional ingredient and hypoglycemic agent	Modulated gut microbiota and improved glycemic profile	(Mo et al. 2024)

**SCFAs**: short‐chain fatty acids; **WHC**: water holding capacity.

## 8 Interactions of Plant Fibers and Macronutrients

Incorporating plant fibers into foods has gained relevance in the food industry due to the growing interest in their functional properties and nutritional benefits (Weaver and Givens 2025). Although the positive effects of dietary fibers on intestinal health and cholesterol reduction are widely discussed, the interactions of these fibers with other nutrients present in food matrices are still little explored (Ndou et al. 2024).

Plant fibers can interact significantly with macronutrients, such as proteins, starches, and lipids, directly affecting structural and textural characteristics and food stability. Such interactions are decisive for the physical‐chemical behavior of products, influencing their final quality and consumer acceptance (Aguilera 2025; P. Yu et al. 2024).

Regarding proteins, interactions with fibers directly impact the digestibility and functionality of foods. A study conducted by Ye and Yu (2024) demonstrated that different types of fibers, such as pectin, cellulose, and lignin, affect casein digestion differently. Pectin reduced protein digestibility through flocculation interactions and hydrogen bond formation, while lignin directly inhibited proteolytic enzymatic activity. Although with a less pronounced effect, cellulose also reduced the release of essential amino acids. These findings highlight the importance of selecting fiber types appropriately in protein formulations, as they significantly influence the formation of protein networks that contribute to the texture of products such as bread and meat analogues (Teixeira et al. 2024).

Fibers also exert a relevant influence on carbohydrates, especially during starch gelatinization. They can compete for water, altering viscosity, gel strength, and starch retrogradation (Sempio et al. 2025). Sahin et al. (2023) observed that the addition of fibrous ingredients to cookies promoted an increase in the product's hardness, attributed to accelerated recrystallization. In addition, insoluble fibers competed with starch for water, influencing the release of sugars during digestion and, consequently, reducing the glycemic index of cookies.

Dietary fiber can interfere with the digestion and absorption of lipids, as well as influence intestinal fermentation. Ndou et al. (2024) demonstrated that the association of fibers such as pectin or cellulose with different types of lipids influences the production of SCFAs—compounds that are beneficial for colonic health—in the intestine. Such interactions not only affect the nutritional profile but also impact the sensory properties, shelf life, and functional performance of foods (Núñez‐Gómez et al. 2023).

Therefore, studying the interactions between dietary fibers and macronutrients is essential for developing functional foods with optimized technological and nutritional properties. Understanding these interactions enables the development of healthier and more effective formulations, aligning with the demands of the modern consumer.

## 9 Conclusions and Future Perspectives

There seems to be a consensus that raw materials are already produced for producing fibrous ingredients, since by‐products generated in agroindustries can be used. Agro‐industrial by‐products are low‐cost raw materials (or none) with great potential for obtaining relevant ingredients. Industrial interest in plant fibers is growing, and they are becoming an increasingly important substitute raw material.

Adding fibers to food products already adds value to them, also granting them the role of a functional food. Dietary fibers present a vast range of options for food uses (both as additives and enriching ingredients). Several studies have demonstrated the feasibility of extraction methods according to the desired fiber objective and the association of methods aimed at improving process effectiveness. Concerning only the final yield is a hasty action since the core of the process is to provide a more efficient method that brings beneficial changes to the fiber under analysis.

Studies on different types of fiber extractions in different plant matrices are increasingly necessary, mainly because they provide references for development and application on a large scale (industrial applications). Fibers from by‐products and co‐products have proven to be highly profitable for food enrichment. Therefore, research on plant matrices is essential for understanding the mechanisms that influence favorable properties and for obtaining better‐quality raw materials.

Future research should focus on interdisciplinary approaches integrating materials science, chemical engineering, and artificial intelligence to optimize fiber recovery, functionalization, and application. For example, machine learning algorithms can be used to model and predict the behavior of fiber extraction and modification systems, reducing experimentation time and increasing process efficiency. At the same time, advanced materials characterization techniques can be combined with chemical engineering to develop new functionalization methods that enhance the functional properties of recovered fibers. Such integrated approaches hold promise for accelerating the transition from laboratory solutions to industrial applications, fostering sustainable innovations in textiles, biomaterials, and biodegradable packaging.

## Author Contributions

**Raquel Martins da Silva Fernandes de Oliveira**: conceptualization, investigation, writing – original draft, writing – review and editing. **Herald Martinho Lino dos Santos**: conceptualization, investigation, writing – original draft, writing – review and editing. **Sibele Santos Fernandes**: conceptualization, investigation, writing – original draft, writing ‐ review and editing, supervision. **Mariana Buranelo Egea**: conceptualization, investigation, writing – original draft, writing – review and editing, funding acquisition, supervision.

## Conflicts of Interest

The authors declare that they have no conflicts of interest.
